# Elabela and Apelin regulate coronary angiogenesis in a competitive manner

**DOI:** 10.17912/micropub.biology.000886

**Published:** 2023-09-14

**Authors:** Syeda A. Madiha, Bikram Sharma

**Affiliations:** 1 Department of Biology, Ball State University, Muncie, Indiana, United States

## Abstract

APJ, a G-protein coupled receptor, regulates coronary angiogenesis in the developing mouse heart. However, the exact mechanism by which APJ regulates coronary angiogenesis from its dual ligands, ELABELA and APELIN, is unclear. Our study show that ELABELA and APELIN both stimulate angiogenic activities such as proliferation and sprouting outgrowth in explant cultures. We found APELIN to be a more robust angiogenic stimulant compared to ELABELA. When explant cultures were stimulated by both ligands together, we found that ELABELA repress the angiogenic activity of APELIN. Collectively, we show that ELABELA and APELIN regulate coronary angiogenesis in a competitive manner.

**Figure 1. APJ signaling from its dual ligands regulates proliferation and angiogenic sprouting of coronary endothelial cells in a competitive manner.**
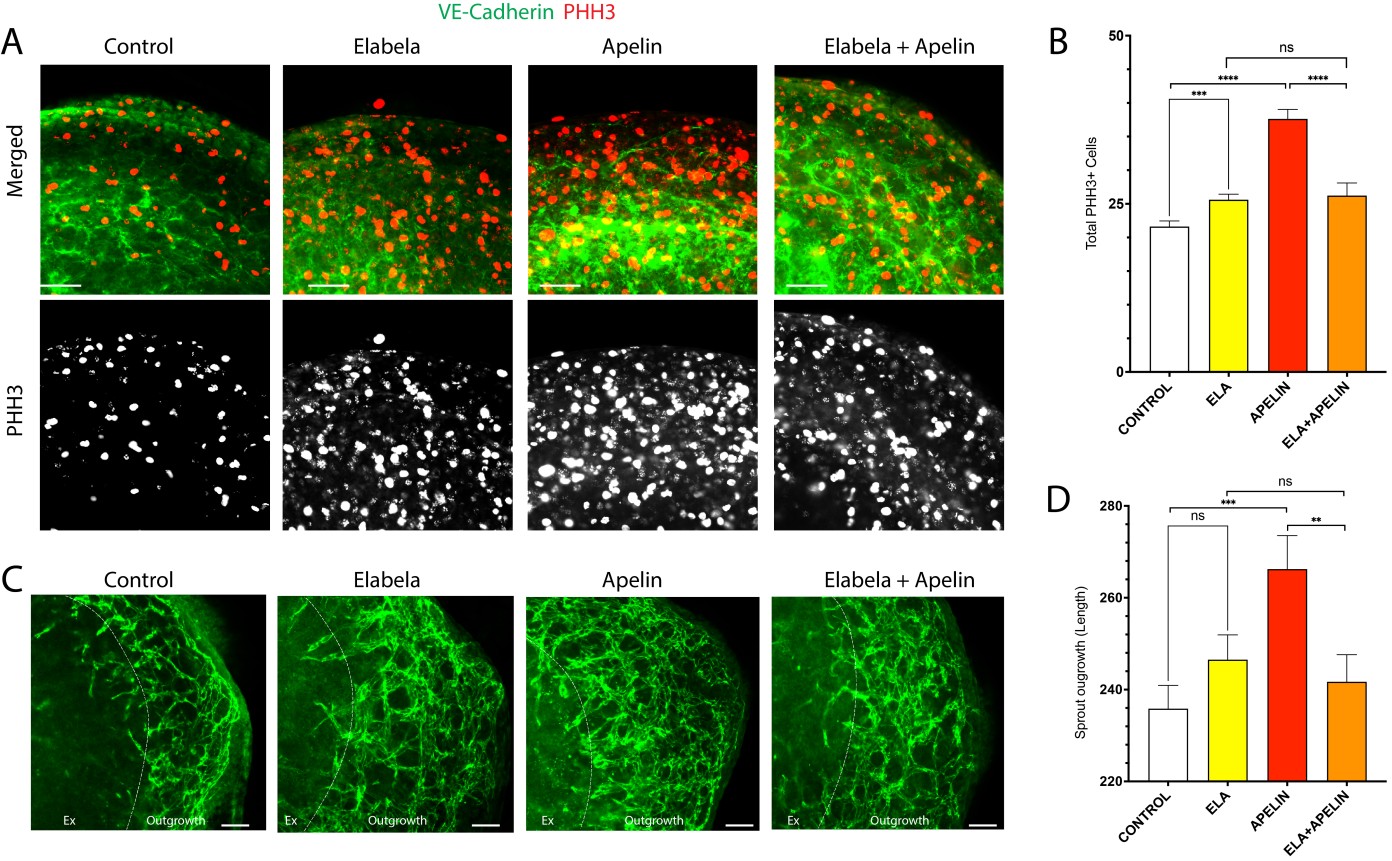
A) Immunostaining of the ventricular explant culture of e13.5 mouse hearts. Cellular proliferation is detected by immunostaining PHH3 (in red), and coronary capillary plexus is stained with VE-Cadherin (in green). B) Quantification of PHH3+ cells in different experimental groups. C) Angiogenic sprouting of ventricular explant culture of e13.5 mouse heart is visualized by immunostaining VE-Cadherin (in green). D) Quantification of sprout length in different experimental groups. Each bar graph represents Mean ± Standard Deviation. **, p < 0.01, ***, p < 0.001, ****, p < 0.0001.

## Description

APJ signaling from its dual ligands, ELABELA and APELIN, plays a major role in cardiovascular development and function (Szokodi et al. 2002; Kuba et al. 2007; Scott et al. 2007; Zeng et al. 2007; Chng et al. 2013; Kang et al. 2013; Helker et al. 2015; Sharma et al. 2017; Kuba et al. 2019) . APJ is known to stimulate coronary angiogenesis in embryonic mouse hearts (Kang et al. 2013; Sharma et al. 2017) . Knockout studies in mice and zebrafish indicated the importance of APJ with its dual ligands, APELIN and ELABELA, on cardiovascular development (Chng et al. 2013; Pauli et al. 2014; Sharma et al. 2017) . Discrepancies in knockout phenotypes of *Apj* and *Apelin* led to the discovery of ELABELA as an alternate ligand of APJ (Scott et al. 2007; Zeng et al. 2007; Chng et al. 2013) . *Apj* and *Elabela* knockout in mouse embryos significantly stunted coronary angiogenesis, whereas *Apelin* knockout increased it (Kang et al. 2013; Sharma et al. 2017) . Therefore, it is still unclear how APJ regulates coronary vessel formation through its dual ligands. This study sought to explore the effects of APELIN- and ELABELA-mediated APJ signaling on the angiogenic activity of coronary vessel formation by assessing coronary endothelial cell proliferation and sprouting outgrowth. These cellular processes are integral aspect of coronary angiogenesis, a process by which coronary vessels grow in developing hearts. To study this, we performed *in vitro* explant culture experiments and stimulated coronary growth in the cultures under various treatment conditions to activate APJ signaling via its dual ligands APELIN and ELABELA.

To determine the role of APJ signaling from its dual ligands, ELABELA and APELIN, on coronary endothelial cell proliferation during coronary angiogenesis, we performed PHH3 staining on *in vitro* explant culture model of coronary angiogenesis as described previously (Large et al. 2020) . We performed whole mount immunostaining on stimulated cultured explants using anti-PHH3 antibody to detect PHH3 expression, which is a marker for proliferating cells. Our results showed a greater number of PHH3+ cells in all three treatment groups (ELABELA, APELIN, and ELABELA+APELIN) when compared to control ( **Figure 1A )** . APELIN treatment resulted in the most PHH3+ cells out of all four groups. Both ELABELA- and ELABELA+APELIN-treated explants show similar amount of PHH3+ cells. We found significantly increased number of PHH3+ cells in explants treated with ELABELA or APELIN when compared to control ( **Figure 1B** ). There was a modest increase in the total number of proliferating cells in ELABELA-treated explants compared to control explants. APELIN, on the other hand, showed a substantial increase in PHH3+ cells when compared to control. APELIN-treated explants exhibited a twofold increase in the total number of PHH3+ cells when compared to control PHH3+ cells. Interestingly, we found significantly decreased number of PHH3+ cells when stimulated with ELABELA+APELIN compared to individual stimulation by APELIN; there was an almost a twofold decrease in PHH3+ cells in ELABELA+APELIN treated groups compared to APELIN treated groups. However, there was no significant difference between individual stimulation by ELABELA and stimulation with ELABELA+APELIN. Our results show that ELABELA and APELIN both stimulate coronary EC proliferation and that APELIN is a much stronger stimulator than ELABELA. However, the effect of APELIN is significantly repressed when combined with ELABELA. Our results suggest that ELABELA negatively interferes with APELIN signaling in regulating coronary angiogenesis.

To determine the role of APJ signaling from its dual ligands, ELABELA and APELIN, on coronary endothelial cell sprouting, we performed anti-vascular endothelial cadherin (VE-Cadherin) staining to visualize angiogenic outgrowth (sprouting) of endothelial cell in explant cultures stimulated with ELABELA and APELIN, individually or in combination. Our results showed longer coronary vessel outgrowth from the explants for all three treatment groups (ELABELA, APELIN, and ELABELA+APELIN) when compared to untreated control explants ( **Figure 1C )** . APELIN treatment resulted in the longest sprouting outgrowth, whereas ELABELA treatment significantly repressed the effect of APELIN on angiogenic outgrowth and resulted in stunted outgrowth compared to individual treatment with either ELABELA or APELIN. We observed a significant, nearly twofold increase of coronary vessel outgrowth in APELIN treatment groups compared to control ( **Figure 1C** ). However, there was no significant difference between control and ELABELA-treated explants even though ELABELA did increased the sprouting outgrowth compared to control. Similar to the proliferation results, combined treatment of APELIN and ELABELA resulted in a significant, nearly onefold decrease in sprouting compared to APELIN treatment. Finally, there was a modest (but not statistically significant) decrease in the sprout outgrowth in ELABELA+APELIN treated explants compared to ELABELA treated explants ( **Figure 1D ).** Collectively, our results show that APELIN stimulates coronary outgrowth (migration), but its effect is repressed by ELABELA, suggesting that ELABELA and APELIN regulate coronary outgrowth in a competitive manner *in vivo* since both the ligands are expressed in the mouse hearts.

ELABELA and APELIN have previously been shown to function through its common receptor APJ in the regulation of cardiovascular function (Ho et al. 2017) and in fluid homeostasis (Deng et al. 2015) . In addition, ELABELA and APELIN loss-of-function studies showed an opposite coronary growth phenotype in mice (Sharma et al. 2017) , indicating that these two ligands potentially compete for signaling through the APJ receptor. Our *in vitro* data in this study show that APELIN elicits a robust angiogenic effect, whereas ELABELA stimulate a moderate angiogenic effect potentially through the APJ receptor. However, our findings suggest that when these two ligands act together on the receptor, ELABELA and APELIN potentially compete for the same binding site thereby moderating signaling through the APJ receptor by dampening the effect of APELIN alone. Therefore, our *in vitro* findings suggest a potential mechanism *in vivo* whereby ELABELA, which is present in the subepicardial surface of the developing mouse heart (Sharma et al. 2017) , modulates APELIN-induced coronary angiogenesis to provide a balanced and moderate growth on the surface (Chen et al. 2014) . In the absence of ELABELA in the myocardium, however, APELIN robustly stimulates angiogenesis (Chen et al. 2014) . Likewise, in the absence of APELIN in the *Apelin* deficient hearts, ELABELA becomes the primary signal on the surface thus resulting in modestly increased coronary growth on the surface as observed in *Apelin* deficient hearts *in vivo* (Sharma et al. 2017) potentially due to the absence of competing signal from APELIN. Collectively, our *in vitro* findings provide a better understanding of how coronary angiogenesis is regulated by APJ receptor through its two ligands, ELABELA and APELIN.

## Methods

All experimental procedures involving animals were conducted in accordance with the Institutional Animal Care and Use Committee (IACUC) of Ball State University.

**Mouse Breeding for Timed Pregnancy**

Male and female CD1 (wild type mice) were bred to produce timed pregnancy in the mouse laboratory at Ball State University. Mice were maintained under standard laboratory conditions (temperature: 25 ± 2º C, humidity: 60 ± 5%, 12-hour dark/light cycle) and fed with a standard laboratory diet and water. Breeding was maintained until the embryos reach the appropriate age for analysis. To determine embryonic age, the morning of vaginal plug detection was designated as embryonic day 0.5 and vaginal plugs were checked two times a day, once in the morning and once in the evening.

**Harvesting Embryonic Hearts for Explant Culture**

Embryos were harvested for experiments on embryonic day e13.5. Pregnant mice were euthanized in CO _2_ chamber and death was confirmed by cervical dislocation. Embryos were dissected from the uterus and placed in pre-chilled 1X sterile phosphate-buffered saline (PBS) solution. The hearts from the embryos were extracted and subjected to explant culture as previously described (Large et al. 2020) .

***In vitro* Explant Culture of Embryonic Hearts**

Isolated hearts were subjected to culture in 24-well plates on top of a round cover glass. Heart explants were placed atop the coverglass carefully at the center of the wells. Explants were cultured at low volume (~200μl of complete media (Microvascular Endothelial Cell Growth Medium (EGM-2 MV)) to keep the culture at air-liquid interface to avoid floating. Cultures were then incubated for 24 hours in 37 ^o^ C, 5% CO _2_ incubator. After the attachment of the explants onto the cover glass, the cultures were subjected to treatments the next day.

**Treatment of Explant Cultures**

Before the treatment, the explants were washed with 1X PBS once for 5 min, starved in basal media (Endothelial Cell Basal Medium (EBM-2) for 3 hours, and washed again with 1X PBS for 5min. Negative control samples were treated with just the basal media, ELABELA groups were treated with the basal media containing 5 μM ELABELA, APELIN groups were treated with basal media containing 5 μM APELIN, and ELABELA+APELIN groups were treated with basal media containing 5 μM ELABELA and 5 μM APELIN. Treatments were prepared at 300 μl total volume per well and were added to the corresponding culture wells. Explant cultures were incubated in the corresponding treatments for 24 hours. After the treatment, the explants were washed once with 1X PBS for 5min, fixed with 4% paraformaldehyde (PFA) shaking in 4 ^o^ C for 15 mins, and washed again with 1X PBS for 5 min before subjecting them to further analysis.

**Immunostaining**

Explant cultures were immunostained with Anti-phospho Histone H3 (PHH3) (Millipore Sigma Lot: 3449056, 1:250 dilution) and anti-VE-Cadherin (VeCad) (BD Biosciences Cat: 550548, 1:125 dilution) primary antibodies. The explant cultures were stained with primary antibodies overnight in 4 ^o^ C shaking, and the antibodies were washed with 1X PBS the next day. Secondary antibodies used were Alexa Fluor 488 goat anti-rat IgG (ThermoFischer Scientific, Cat: A11006) and Alexa Fluor 555 goat anti-rabbit IgG (ThermoFischer Scientific, Cat: A21429) both at 1:250 dilution. The cultures were immunostained in secondary antibodies overnight in 4 ^o^ C shaking covered with aluminum foil to prevent photobleaching. The next day the explants were washed with 1X PBS (3 times 5 min each). After wash steps were complete, explants in cover glass were mounted onto the glass slides. Mounting media containing DAPI (mounting medium with nuclear marker DAPI, VECTOR Laboratories, Cat: H-2000-10) was added onto the explants. Coverslips were carefully added to cover the samples on the slides. Cover glass was sealed with clear nail polish. These slides were imaged using epifluorescent/confocal microscope to collect data. Images were analyzed using Fiji, an image analysis software.

**Microscopy**

Images of the explants were taken using an inverted confocal microscope (LSM 5 PASCAL). Each antibody was detected at different wavelengths with PHH3 staining in 543nm, VeCad in 488nm, and DAPI in 405nm. Multiple fields of view (FOVs) were imaged to capture the whole heart (average of 6 FOVs per explant). Each FOV was imaged at 5 μm interval. In average, 12 optical slices were obtained per FOV.

**Data Analysis**

Cellular **proliferation** was analyzed by first making a Z stack projection with maximum intensity of all the z-stack optical slices for each FOV of the explant using the Fiji software and then counting PHH3+ cells within multiple FOVs that is representative of the whole heart. Three 200 x 200 μm squares were drawn at spatially identical regions within each FOV (one on the top, one in the middle, and one towards the bottom) for each FOV image of the explant and then the PHH3+ cells within those three squares were counted and used for statistical analysis. The coronary endothelial sprouting outgrowth was analyzed by first making a z stack projection with maximum intensity using the Fiji software. The outgrowth of coronary sprouts from the explant was then measured (visualized by VE-cadherin staining of coronary endothelial cells) in the explant cultures. Three equally distant sprout lengths were measured for each images representing a FOV. Images capturing multiple FOV are quantified to represent the whole heart.

**Statistical analysis**

For each experiment, data were collected from 3 biological repeats and samples in each experimental conditions were run with at least three replicates. Significant differences between the two experimental groups were analyzed by t-test (unpaired t-test with Welch’s correction) using Prism 9.0 statistical software. P < 0.05 is considered statistically significant. Data are represented in bar graphs as Mean ± SD.
